# Avian myeoloblastosis virus (AMV): only one side of the coin

**DOI:** 10.1186/1742-4690-5-49

**Published:** 2008-06-16

**Authors:** Bernard Perbal

**Affiliations:** 1 Department of Dermatology, University of Michigan Medical School, 1150 W. Medical Center Dr., Medical Science I, Room 6447, Ann Arbor, Michigan 48109-0609, USA

## Abstract

For many years, scientists and suppliers have refered to AMV-RT as the reverse transcriptase produced by the Avian Myelobalstosis Virus. This manuscript briefly reviews the molecular basis for biological dependence of AMV for the envelope and RT proteins that are produced by its natural helper the Myeloblastosis Associated Virus (MAV). Because the wide use of the term «AMV RT» obscures scientific facts, it is worthwhile to clarify this issue for the scientific community, especially for younger scientists who might not be aware of the functional relationships that exist between these two viruses.

## Background

The Avian Myeloblastsis Virus (AMV) is well known among molecular biologists because AMV stocks have been used over several decades as one of the major source of avian Reverse Transcriptase (RT). Originally identified from Rous Sarcoma Virus (RSV) [1] and Rauscher Leukaemia virus (RLV) [2], RT was also found to be an essential enzyme in the life cycle of other retroviruses and RNA viruses. The identification of RT with RNA-dependent DNA polymerase activities has enabled new developments in many areas of Biology.

In the life cycle of retroviruses, RT transcribes the single stranded RNA genome into double stranded proviral DNA, which is integrated into host DNA and from which viral progeny is made. This process requires three steps, each catalyzed by the RT enzyme: synthesis of DNA from the viral RNA template, hydrolysis of the RNA strand (RNAseH activity), and syntheisis of double stranded DNA from the single stranded DNA template.

Although it is known for a long time that the rate of genomic mutation is particularly high among animal retroviruses, this aspect has drawn a lot of attention in the case of HIV for which the very efficient multiplication of the virus *in vivo *is the source of extreme diversity [3].

Mutations that are identified in the viral genome can theoretically be introduced at any of the various steps that lead to proviral DNA either during the synthesis of the minus-strand cDNA copy of the genomic RNA template, during the synthesis of the second DNA strand which are both performed by reverse transcriptase, or during the transcription of the proviral genome that is performed by RNA polymerase II. When the relative contribution of each replication stage to the retroviral mutation rate was studied with defined templates [4] it was established that mutations occur mainly at early stages, with a nearly similar mutation rate for the two polymerizations catalyzed by reverse transcriptase. However, these conclusions are weakened by the fact that the error rates measured in vitro are approximately an order of magnitude higher than the overall error rates measured in a single cycle of infection in cultured cells.

The enzyme isolated from AMV-infected cells is composed of two structurally related sub-units designated α and β (molecular weights of 65,000 and 95,000 daltons, respectively) assembled into an α β holoenzyme which is generated by proteolytic cleavage of a minor less active β β precursor [5,6]. The α subunit of the enzyme has both the RNA-directed DNA polymerase activity specific to reverse transcriptase and an RNase H activity. The RNase H activity is associated with a 24,000-dalton fragment and is generated by proteolytic cleavage of the α subunit. The polymerase activity of reverse transcriptase is dependent on the presence of a primer and a template [7,8].

The AMV RT exhibits a greater processivity than the murine RT and retains activity at higher temperatures than the murine RT because of a higher intrinsic thermal stability [9]. For these reasons, and because the use of RT in fundamental and applied biomedical fields has become widespread by the introduction of PCR, one of the most commonly used commercial RT is derived from AMV stocks.

## The viruses

The avian myeloblastosis virus (AMV) is an alpha retrovirus responsible for acute myeloblastic leukemia (AML) when injected in ovo, or in newly hatched chickens [10]. Early in vitro dose response experiments indicated that the production of virion with leukemogenic potential required a double infection with AMV and a helper virus [11]. The AMV strains that are used and commercially available, are derived from the orignal BAI strain A purified from chicken leukemic plasma [12]. Leukemic plasma containing the BAI strain has been widely distributed for many years by Life Sciences Inc., in Florida which has been the official provider of national agencies in the USA. The Standard AMV-S BAI strain is a complex mixture of viruses that also includes two helper viruses in addition to AMV. The helper viruses Myeoloblastosis Associated Virus (MAV) contained in AMV-S belong to two different serological subgoups (type1 and type2, also called A and B). Both of them are oncogenic [13].

Both MAV-1 and MAV-2 are replication-competent retrovirus with a 7.7 kb genome that encodes seven structural gag proteins (the viral proteins of the matrix (p19, MA), capsid (p27, CA) and nucleocapsid (p12, NC), a protease (p15, PR) and the p1, p2, and p10 proteins of unknown functions), four pol proteins (95 kDa b and 63 kDa a subunits of the reverse transcriptase, the 32 kDa integrase and a 4.1 kDa peptide of unknown function), and two structural env glycoproteins [the gp85, surface (SU) and gp37 transmembrane (TM) polypeptides]. Both AMV and MAV have unique 3' regions (U3) which contain sequences involved in viral transcription. The U3 region of MAV and AMV is very similar and does not resemble other retroviral U3 regions, however, the U5 region of these viruses differs from that of Rous sarcoma virus (RSV) by only a few base mismatches.

Early studies have established that plaque-purified MAV of both types could induce predominantly osteogenic osteoblastomas and nephroblastomas and less frequently, visceral lymphoid leukosis [reviewed in ref [13]]. Two purified strains of MAV2 were reported to have slightly different oncogenic properties. The MAV2(O) induced 80% osteopetrosis and 20% nephroblastomas whereas the MAV2(N) induced 80% nephroblastomas and 30% osteopetrosis [13]. Differences in T1-resistant oligonucleotides maps of viral RNAs established that the MAV2(O) and MAV2(N) strains were genetically distinct.

The acute transforming activity of AMV that results from the oncogenic properties of v-myb [14] is giving rise to AML, an infected animals die long before they can develop tumors that are induced by the MAV component present in the viral stocks.

Molecular cloning of MAV-1 and MAV-2 proviral genomes permitted to better define the oncogenic properties of these viruses and established that genetically pure MAV2 strains retained their dual pathogenic potential, when injected in Brown Leghorn chickens (Edinburgh strain, gs+, chf+, V-), therefore indicating that it did not result from a mixed population of viruses [15]. The substitution of a single amino acid in the MAV2(O) TM env protein was proposed to abolish its capacity to induce osteopetrosis [15].

The MAV1 provirus that was cloned from a library of leukemic chicken myeloblast DNA [16] was shown to be of the N type and induce 100% nephroblastomas when injected either intraveneously in 12-day embryos or intraperitoneally to day-old chicken [17]. The highly restricted oncogenic properties of MAV1(N) made it a unique tool to study nephroblastoma development at a molecular level and has led to the discovery of nov/ccn3, which encode a member of the emerging CCN family of cell growth regulators [18]. Nucleotide sequencing of the MAV-1 and MAV-2 proviral genomes identified MAV-1 as the natural helper of AMV [16] and established that AMV derived from MAV [19].

The AMV proviral genome that had also been cloned from peripheral blood leukemic myeloblasts [20] proved to be significantly different from MAV.

**Figure 1:**
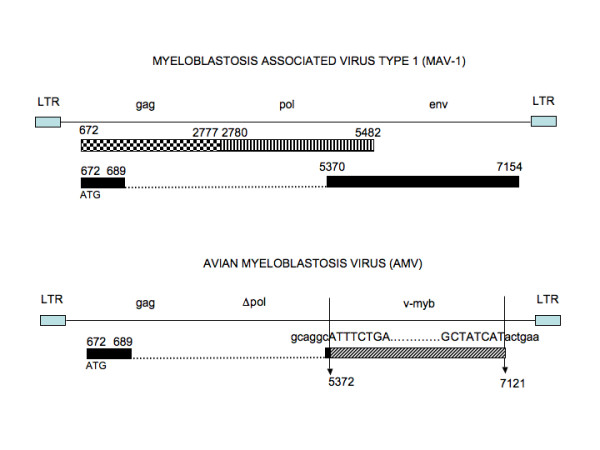
**Schematic organization and expression the MAV and AMV proviral genomes**. The MAV RNA genome that is contained in infectious particles is retrotranscribed by RT and viral proteins are expressed from the proviral double stranded DNA copy of the viral genome, which is integrated into the host genome. The structural glycoproteins (MA, p10, CA, NC, PR) and reverse transcriptase (POL) are produced by post translational processing of the gag-pol polyprotein precursor, which is expressed in the form of a bicistronic message (n672-n2777, n2780-n5482) from the proviral genome. The envelope protein is made from spliced mRNA species that join 17 aminoterminal amino acid residues to the env ORF that begins at nucleotide 5370 of MAV and overlaps the 3' end of the pol gene, encoding the integrase activity (IN). MAV infected cells contain the viral RNA genome, and both the subgenomic gag-pol precursor and env mRNAs. Cell-derived v-myb oncogenic sequences are transduced by AMV. Recombination of the truncated c-myb c-DNA copy with the MAV proviral genome occurred within the pol and env genes of MAV at nucleotides 5372 and 7121, respectively in the proviral DNA of MAV-1. The env splice acceptor localized upstream to the 5' proximal recombination point in MAV DNA, is used to join the v-myb ORF to the 17 aminoterminal residues of the gag protein. As a consequence of the v-myb integration the AMV is replication defective because it lacks the gag and pol proteins. Only the genomic viral RNA and the v-myb RNA are expressed from the proviral AMV. The high degree of conservation between the pol coding sequences which are contained in the MAV genome and the portion of pol sequences contained in the AMV genome, suggest that recombination between AMV and MAV may occur at this level, and result in the production of pol proteins from both genomes. However, this has not been demonstrated as yet. *top*: MAV proviral genome. Solid boxes represent the LTR sequences which are generated during the process of proviral DNA synthesis and integration. The gag-pol polyprotein precursor comprises gag sequences (stipled box) and pol sequences (striped box). Proteolytic cleavage of the precursor occurs at position corresponding to codon 2777–2780. The two black boxes represent the coding sequences of the envelope protein that are joined by splicingw which occurs between the gag splice donor at nucleotide 689 and the splice acceptor at position 5370 in the pol sequences. *Bottom*: proviral AMV genome. The v-myb coding sequences are made of a part of gag (black box) and c-myb derived sequences (hatched box). Arrows indicate the points of recombination of the c-myb derived sequences in the MAV genome.

Molecular cloning of the v-myb oncogene [21] and nucleotide sequencing of both MAV and AMV proviral genomes established that in AMV, the insertion of v-myb oncogenic sequences resulted in the deletion of the entire env gene and of the 3' portion of the pol [22] encoding the integrase (IN). The lack of envelope and integrase accounts for the defectiveness of AMV [reviewed in [23]] (figure 1).

Myeloblasts transformed by purified AMV in the absence of MAV released defective virus particles which contained gag proteins but lacked both the env glycoprotein and transcriptase [24], while cells infected by purified MAV do not produce sufficient virions to make worhtwhile purifying MAV particles for purification of RT.

Upon infection of target cells with both MAV and AMV, MAV provides in trans the envelope and integrase proteins that permits i) the production of MAV virions ii) the production of pseudotype-1 AMV virions and iii) the integration of the AMV proviral genome in the host DNA. Myeloblasts that are transformed by the v-myb protein proliferate very actively and produce very high titers of virions which are a mixture of AMV and MAV, with a large excess of the helper virus that encode a fully active RT.

## Discussion

From what is briefly summarized above, it comes that the RT which is purified from AMV stocks does not originate from AMV but for the helper virus MAV.

Although both transcriptase and RNAseH activities are encoded by both viruses, the recombination of v-myb sequences in AMV interferes with the production of subgenomic RNA transcripts encoding these two proteins, as shown by analysis of viral progeny in purified AMV-infected cells [24]. The presence of AMV is required to bring the v-myb oncogene that drive intense proliferation of transformed myeloblasts that express at the same time the MAV-encoded proteins. Since recombination between retroviral genomes has been documented in other systems, one might argue that recombination occurs between MAV and AMV genomes in infected cells, leading to the production of RT by AMV. However, this is very unlikely as juged by the nucleotide sequences established from several independent isolates of MAV and AMV that did not show any variations with respect to each other.

In spite of these published data, most companies still claim to provide RT isolated or cloned from pure strains of AMV. This erroneous understanding of the source of RT has been propagated throughout the scientific community.

## Conclusion

What was acceptable in the early days of retrovirology because of lack of knowledge should be corrected.

We as educators and scientists have the responsibility to use accurate terminology and disseminate validated concepts. For these reasons, scientists and the numerous RT suppliers should consider that AMV-RT is a misnomer and inform those who are unaware of this particlular situation.

There are many other examples of genes, proteins, or other biochemical agents that have been given a name, which is later found to be scientifically inaccurate or misleading. In some cases new knowledge has prompted a name change to allow persons to use language that is more accurate. Whether scientists and suppliers might consider referring to RT as "AMV/MAV RT" instead of "AMV RT" in lectures, manuscripts and books to avoid unnecessary confusions might be worth considering.
